# Field evaluation of piglet vaccination with a *Mycoplasma hyopneumoniae* bacterin as compared to a ready-to-use product including porcine circovirus 2 and *M. hyopneumoniae* in a conventional French farrow-to-finish farm

**DOI:** 10.1186/s40813-017-0077-y

**Published:** 2018-01-18

**Authors:** Didier Duivon, Isabelle Corrégé, Anne Hémonic, Martial Rigaut, David Roudaut, Rika Jolie

**Affiliations:** 1 MSD Santé Animale, 7, rue Olivier de Serres - Angers Technopole, C.S. 17144, 49071 Beaucouzé cedex, France; 2 IFIP, La Motte au Vicomte, 35650 Le Rheu, France; 3 MSD Animal Health, 2 Giralda Farms, Madison, NJ 07940 USA

**Keywords:** PCV2, *Mycoplasma hyopneumoniae*, Vaccine, Randomized controlled field trial

## Abstract

**Background:**

A controlled randomized trial was performed on a well-managed conventional French 180-sow farm. The trial compared the growth performances of piglets vaccinated at weaning (single shot) either with a commercial monovalent *Mycoplasma hyopneumoniae* bacterin vaccine or with a commercial bivalent vaccine (Porcilis® PCV M Hyo) against *M. hyopneumoniae* and porcine circovirus 2 (PCV2). The farm’s porcine reproductive and respiratory syndrome status was stable, and most diseases (enzootic pneumonia, atrophic rhinitis, post-weaning multisystemic wasting syndrome) were controlled by routine vaccination.

**Results:**

During the post-weaning phase, the growth performances of the piglets vaccinated with the bivalent vaccine were not significantly different from those vaccinated with the monovalent vaccine. However, during the fattening phase the group vaccinated with the bivalent vaccine had a significantly improved ADG (+34 g/d, *p* = 0. 047), resulting in a 5-day earlier shipment to slaughter. The group also had a shorter and lower PCV2 load in serum during the fattening period, and an improved lung lesions score. In both groups, three pigs died during the peak PCV2 viraemia (16–23 weeks of age). Immunohistochemistry of the lymph nodes showed that in the group vaccinated with the bivalent vaccine, none of these pigs had PCV2-like lesions, while 2 out of the 3 from the other group did. Results suggest that the added PCV2 valence in the vaccination protocol helps countering the negative impact of subclinical PCV2 infection on growth. The calculated return on investment of the added PCV2 vaccine valence was €1.7 extra revenue per slaughtered pig (€ 39 additional revenue per sow and per year), despite the fact that the cost of the bivalent vaccine was higher than the monovalent *M. hyopneumoniae* vaccine.

**Conclusion:**

In this healthy conventional sow farm, the combined *M. hyopneumoniae* and PCV2 vaccination was efficacious, convenient to administer and profitable.

## Background

Subclinical Porcine Circovirus type 2 (PCV2) infection is reported to be the most common form of PCV2 infection worldwide [1]. The only observed manifestation associated with this subclinical infection is a decreased average daily gain. Its diagnostic relies on the individual pig: absence of overt clinical signs, no or minimal histopathological lesions in tissues (mainly lymphoid) and low amount of PCV2 in few (lymphoid) tissues [1]. Since the availability of commercial PCV2 vaccines, veterinary practitioners have observed an improvement of growth performances, even in herds with no overt clinical signs of PCV2-associated diseases. Such field observations have been reported in Canada [2], the UK [3], Spain [4], Germany [5] and Switzerland [6]. Information on the cost of this condition is scarce, although the British report concludes that, at farm level, the economic impact of PCV2 can be mainly attributed to subclinically PCV2-infected pigs [3].

The objective of this trial was to assess the impact on growth performance in pigs of PCV2 vaccination under French farming conditions. In Western France (Brittany), Porcine Respiratory Disease Complex (PRDC) has been extensively studied; its main infectious risk factors are *Mycoplasma hyopneumoniae*, Porcine Reproductive and Respiratory Syndrome Virus (PRRSV) and the Swine influenza virus (SIV) H1N1, while PCV2 is less frequently identified [7]. However, an Austrian study of over 254,000 slaughtered pigs demonstrated that finishers raised on farrow-to-finish farms and that had not been vaccinated against PCV2 were at significantly higher risk of presenting pneumonia post-mortem than vaccinated fatteners from finisher farms [8]. Interestingly, Austria and Brittany have similar pig farm size and production systems.

## Methods

A field trial was carried out on a French 180-sow farrow-to-finish farm, managed by the French Pork and Pig Institute (IFIP, Brittany) in order to evaluate a commercial bivalent vaccine that includes PCV2 and *M. hyopneumoniae* (Porcilis® PCV M Hyo) at weaning, and to compare it to a commercial monovalent one-shot *M. hyopneumoniae* bacterin vaccine.

### The farm

The farm was managed under a strict 7-week all-in all-out system. Its health status was favourable: no active PRRSV circulation (high-parity sows are the only seropositive animals in the herd) and low SIV circulation post-weaning. Both enzootic pneumonia and atrophic rhinitis were present. All these pathogens were controlled through vaccination; no in-feed antibiotic supplementation was provided to the piglets post-weaning. Previous lung scoring indicated an average score of 2.3 out of 28, based on the scoring system according to Madec [9]. Its zootechnical and reproductive parameters place the farm among the top third of French farrow-to-finish farms.^1^

### Piglet preparation

In a 24-sow batch, the otherwise routinely administered PCV2 booster vaccination was omitted, in order to avoid masking the potential effect of piglet PCV2 vaccination by dam vaccination [6]. All piglets born on this farm are individually identified and are weaned at 28 days of age. All piglets from this batch were individually weighed on day 27.

### Group allocation of piglets

Within each litter, piglets were paired accounting for weight and sex; when no pair was available within a litter, the dam’s parity and litter size were taken into consideration when pairing piglets of different litters. Within each pair of piglets, the first piglet was randomly allocated to either vaccine group and the second was allocated to the other vaccine group: the bivalent Porcilis® PCV M Hyo (MycPCV group) or a monovalent *M. hyopneumoniae* bacterin vaccine (Myc group). Both vaccines are based on the same *M. hyopneumoniae* vaccine strain (strain J).

### Room/pen housing

During the entire trial, piglets from different vaccination groups were never mixed in the same pen.

**Fig. 1:**
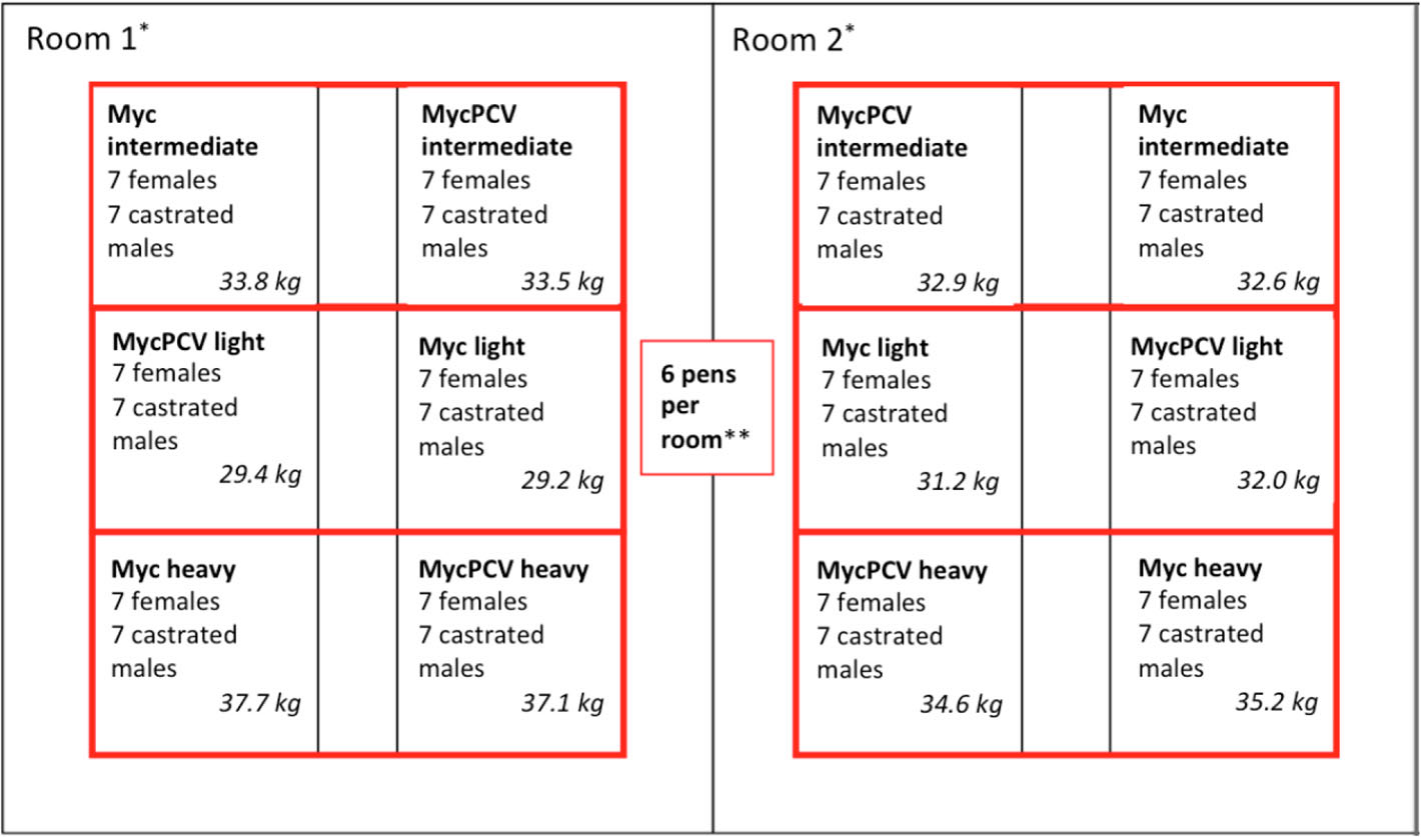
Allocation of groups of pigs in fattening rooms, sorted by weight (three classes). *Animals of each pair (one in the MycPCV group and the other in the Myc group) were placed in adjoining pens. **The average pigs’ live-weight at the start of fattening is mentioned for each pen

At post-weaning, vaccinated piglets were placed in two identical rooms of 6 pens each, with 20 piglets per pen. All pens were identical in design and equipment (with the exception of their symmetry for the corridor, see Fig. 1), with a freely accessible feed trough.

After 6 weeks in post-weaning, piglets were individually weighed and transferred to the 2 adjacent fattening units. Again, all pens were identical in design and equipment, with an automatic feeding station. However, to comply with EU animal welfare regulation on stocking density, each fattening pen could only contain 14 pigs. Due to this constraint, only 84 out of the 120 pairs of piglets could be transferred to the on-site fattening unit.

To gain access to the automatic feeder, pigs have to push a door which lighter pigs (<20 kg live-weight) are not strong enough to open. As a consequence, only the pairs of piglets for which both individuals weighed over 20 kg and were healthy were transferred to fattening.

Pigs that were not doing well (arthritis, hernia…) or single pigs (death of the other pig in the pair) were discarded.^2^ Pigs entering the fattening rooms were sorted for weight (three weight classes, see Fig. 1). Each pen only contained piglets from the same treatment group; in each room, MycPCV and Myc pens were alternated. Automatic feeding stations recorded individual feed intake; feed was provided ad libitum.

All animals were individually weighed at regular intervals, starting 10 days after entering the fattening rooms to allow for adaptation to the automatic feeding system: at 14, 17, 20, 23 weeks of age and then every other week until slaughter.

### Health monitoring

The animals were visited twice daily. All dead animals were removed from their pen for post-mortem examination. When the cause of death could not be ascribed to any other than a PCV2-related disease, mesenteric and inguinal lymph nodes were sampled, identified and frozen. Unthrifty pigs were humanely euthanized, and necropsied following the same protocol.

Blood was sampled from 18 pairs of pigs at 5 weeks of age, and from 12 pairs out of these 18 at 8 weeks of age. The same 12 pairs were repeatedly sampled at 12, 16, 20 and 23 weeks of age. Tests were performed at the Laboratory for Diagnostic Solutions Intervet Boxmeer, the Netherlands, with the IDEXX *M. hyo* Ab Test, the Alphalisa PCV type 2 (*M. hyopneumoniae* and PCV2 serology, respectively), and with an in-house quantitative PCR for PCV2 genome in serum (detection limit of 10^2.9^ genome copies per ml serum; quantification limit of 10^3.4^ genome copies per ml serum).

### Lung checks

Lung checks were performed by the same operator, using the lung scoring system according to Madec [9].

### Pathology

Frozen lymph node tissues were sent to the diagnostic laboratory (Labocea 22, Ploufragan, France). They were thawed and prepared for pathological examination. PCV2-like histopathological lesions were scored in compliance with current terminology [10].

### Statistical analysis

Normality of variables was assessed with the Shapiro-Wilk test. A 5%-threshold was selected for the designation of a statistically significant difference. All statistical analyses were performed with SAS (SAS Institute Inc., Version 9.02). For fattening/finishing performances, the experimental unit was the pen. For clinical and pathological observation/scoring, the experimental unit was the individual pig. When a pig died, its paired piglet was also withdrawn from the data analyses.

## Results

One hundred and twenty pairs of piglets (58 females, 62 castrated males) were vaccinated at 32 days of age. The body weight of the pigs at birth and weaning did not differ significantly between vaccine groups (variance analysis). The post-weaning average daily weight gain (ADG) did not differ significantly between the MycPCV (+511 g/d) and the Myc groups (+513 g/d, multifactorial variance analysis).

Out of the 84 pairs of piglets that entered the fattening rooms, 70 pairs made it to the slaughterhouse. The analysis of the performances was restricted to the data collected for these 140 pigs (see Table 1).

**Table 1:** Performance of the 70 pairs of pigs that remained healthy over fattening

Variable	Myc group^a^				MycPCV group^b^				Difference	*P* value
	Average	Standard deviation	Mini	Maxi	Average	Standard deviation	Mini	Maxi		
Birth weight (kg)	1.5	0.3	0.9	3.0	1.6	0.3	0.8	2.4	0.0	>0.05
Weaning weight (kg)	9.0	1.4	5.3	12.7	9.0	1.4	6.0	12.5	0.0	>0.05
Weight at end of post-weaning (kg)	33.1	2.9	28.0	41.2	33.0	2.5	28.0	38.4	−0.1	>0.05
Post-weaning ADG (g/d)	512.8	56.7	375.5	653.0	511.1	49.6	395.7	634.0	−1.7	>0.05
Weight at start of fattening (kg)	37.2	3.4	30.2	46.6	36.4	3.3	28.4	43.5	−0.9	>0.05
Live-weight at slaughter (kg)	120.6	6.0	105.6	140.6	119.1	4.2	108.3	131.6	−1.5	>0.05
Fattening ADG (g/d)	850.5	85.3	615.9	1043.6	884.5	78.4	626.5	1023.9	33.9	=0.047
Fattening FCR	2.81	0.21	2.41	3.30	2.75	0.21	2.40	3.16	−0.06	>0.05
Age at slaughter (d)	182.5	11.6	161.0	198.0	177.7	9.5	161.0	198.0	−4.7	=0.049
Carcass yield^c^	0.8	0.0	0.7	0.8	0.8	0.0	0.7	0.8	0.0	>0.05
Lean %^c^	60.1	2.2	54.7	65.1	60.1	2.5	54.7	65.3	0.0	>0.05

^a^Six piglets from the Myc group died during fattening

^b^Ten piglets from the MycPCV group died during fattening. There was no statistical difference between the numbers of dead piglets in each group (exact Fischer test, *p* > 0.05)

^c^Data could be collected at slaughterhouse for 64 pairs of pigs

Pigs in the MycPCV group had an average + 34 g/d higher ADG over the fattening period than pigs in the Myc group; this difference was significant (*p* = 0.047). The feed conversion rate (FCR) and feed intake (data not shown) did not differ significantly between both groups over the fattening period (although the FCR was numerically lower in the MycPCV group, see Table 1).

**Fig. 2:**
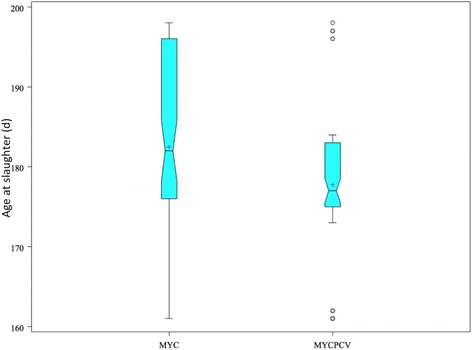
Whiskers plot of the distribution of ages at slaughter for the 70 pairs of pigs

Pigs in the MycPCV group were nearly 5 days younger at slaughter than Myc pigs (p = 0.049). After 186 days of age, 22 fatteners remained in the Myc group, while only 5 remained in the MycPCV group. Batch homogeneity was evaluated by comparing the distribution of the average, standard deviation and coefficient of variation of live-weights at first shipment to slaughter, the ages at 65 kg and slaughter age (data not shown). No significant difference in batch homogeneity was found, although slaughter age appeared to be more homogeneous in the MycPCV group (see Fig. 2). Carcase parameters did not differ significantly between groups (64 pairs evaluated).

*M. hyopneumoniae* antibody levels (S/P ratio) did not differ significantly between groups (non-parametric Mann-Whitney test), with the exception of 23 weeks of age (higher levels in MycPCV vs. Myc, *p* < 0.05), which might reflect a slower decay of post-vaccination *M. hyopneumoniae* antibody levels [11]. Both groups had otherwise similar serological profiles, with low antibody levels on weeks 5–8 (S/P ratio < 0.2, data not shown), increasing levels on weeks 8–12 (peak S/P ratio at 0.4) at and a continuous decrease afterwards.

**Fig. 3:**
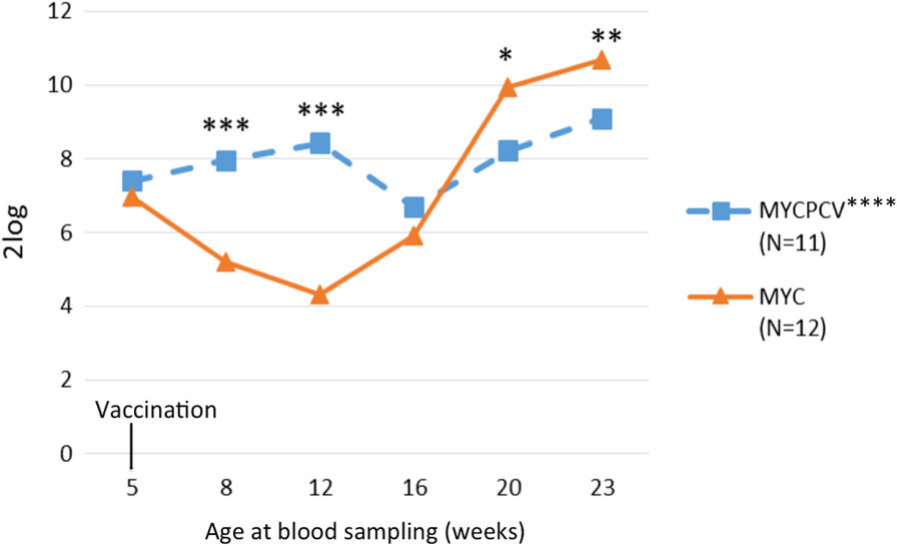
PCV2 serological profiles of the 12 pairs of pigs sampled during the trial. * *p*<0.05; ** *p*<0.01; *** *p*<0.001; **** One sample in the MycPCV group could not be analysed

**Fig. 4:**
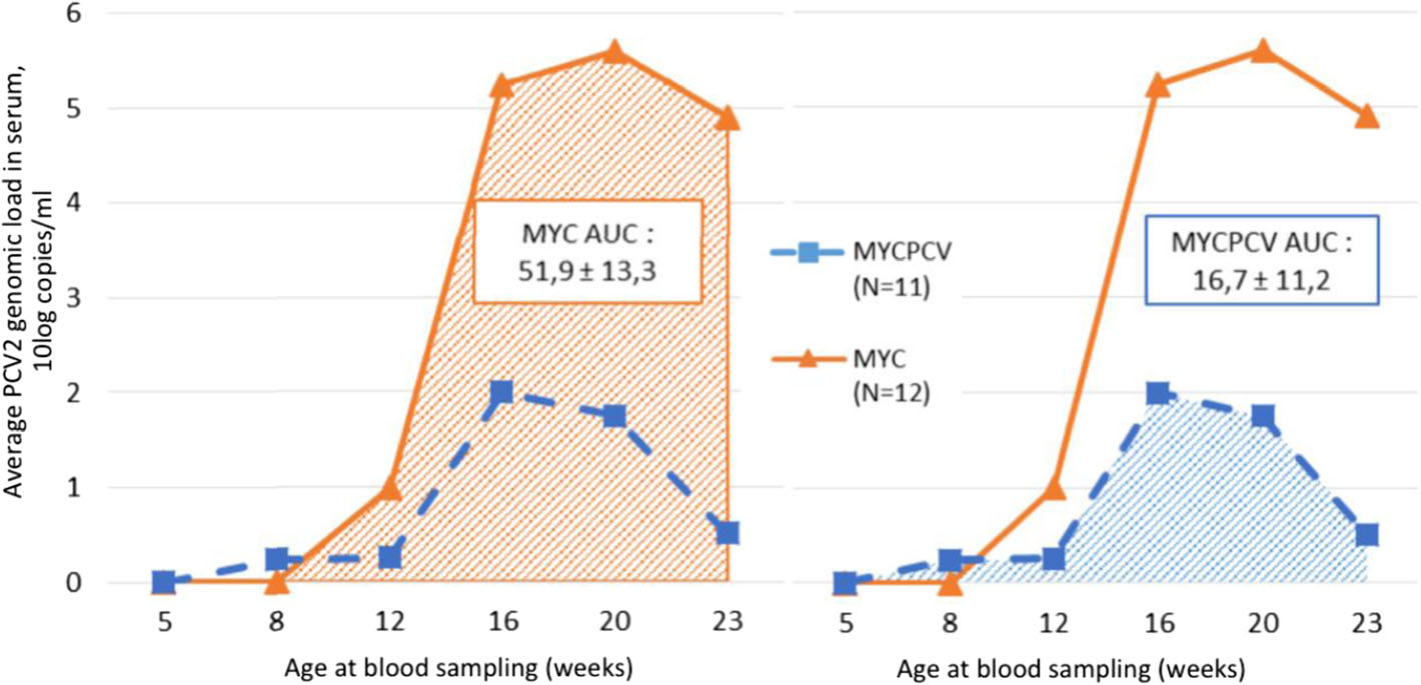
Average PCV2 genomic loads in serum of 12 pairs of pigs from both vaccination groups. (One sample in the MycPCV group could not be analysed)

The PCV2 serological profiles differed greatly between groups (see Fig. 3). The Myc group piglets presented a clear decay in maternal PCV2 antibodies up to week 12. In both groups, the PCV2 genome could not be measured in the serum before week 12. A sharp increase in genomic serum loads was observed in week 16 in the Myc group (see Fig. 4), with a slow decline of the mean PCV2 genome load over the following weeks (10^5.2^, 10^5.6^ and 10^5.0^ in weeks 16, 20 and 23 respectively). In contrast, pigs in the MycPCV group had significantly lower mean viraemia levels on weeks 16 and 20, and appeared to have cleared the infection by week 23 (10^1.7^, 10^2.0^ and 10^0.5^ on weeks 16, 20 and 23 respectively). This difference is even more striking when comparing the areas under the curve (AUC) (normal distribution, Shapiro-Wilk test, *p* = 0.4413): 51.19 ± 13.33 in the Myc group and 16.70 ± 11.20 in the MycPCV group (Student t test, *p* = 8.583 10^−7^).

**Fig. 5:**
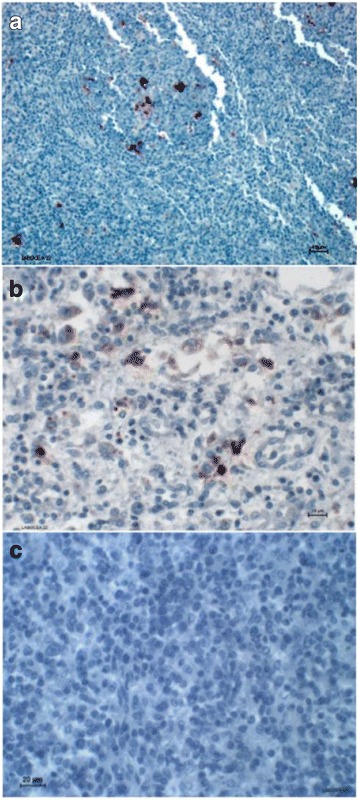
Immunohistochemistry results on lymph nodes of pigs dead during the fattening period (scale bars: A, 40 μm; B, 15 μm; C, 20 μm). **a**. Weakly PCV2-positive immunohistochemistry of a lymph node from a dead pig of the Mhyo group, whose pathology presented a marked granulomatous lymphadenitis, highly suggestive of PCV2-associated lesions. **b**. PCV2-positive (+ and ++) immunohistochemistry stains of a lymph node from a dead pig of the Mhyo group, whose pathology presented a moderate lymphadenitis, suggestive of PCV2-associated lesions. **c**. No obvious PCV2-positive IHC in a lymph node from a pig dead of gastric ulceration in of the Mhyo group

When the viremia results were obtained, the supervising veterinarian, in order to confirm whether the effect of PCV2 infection was observable in the tissues of the dead pigs (no overt clinical sign of PCV2-associated disease had been observed during the trial), sent the biological samples that had been stored in proper conditions (frozen) to the diagnostic laboratory. Three pigs in each group had died over the period of the PCV2 viremia peak (weeks 16–23). PCV2-like histopathological lesions, and PCV2 antigen in the lesions based on immunohistochemistry, were observed in the lymph nodes of 2 out of the 3 pigs from the Myc group that had died, while no such lesion/marking was observed in any of the 3 dead pigs from the MycPCV group (see Fig. 5).

**Fig. 6:**
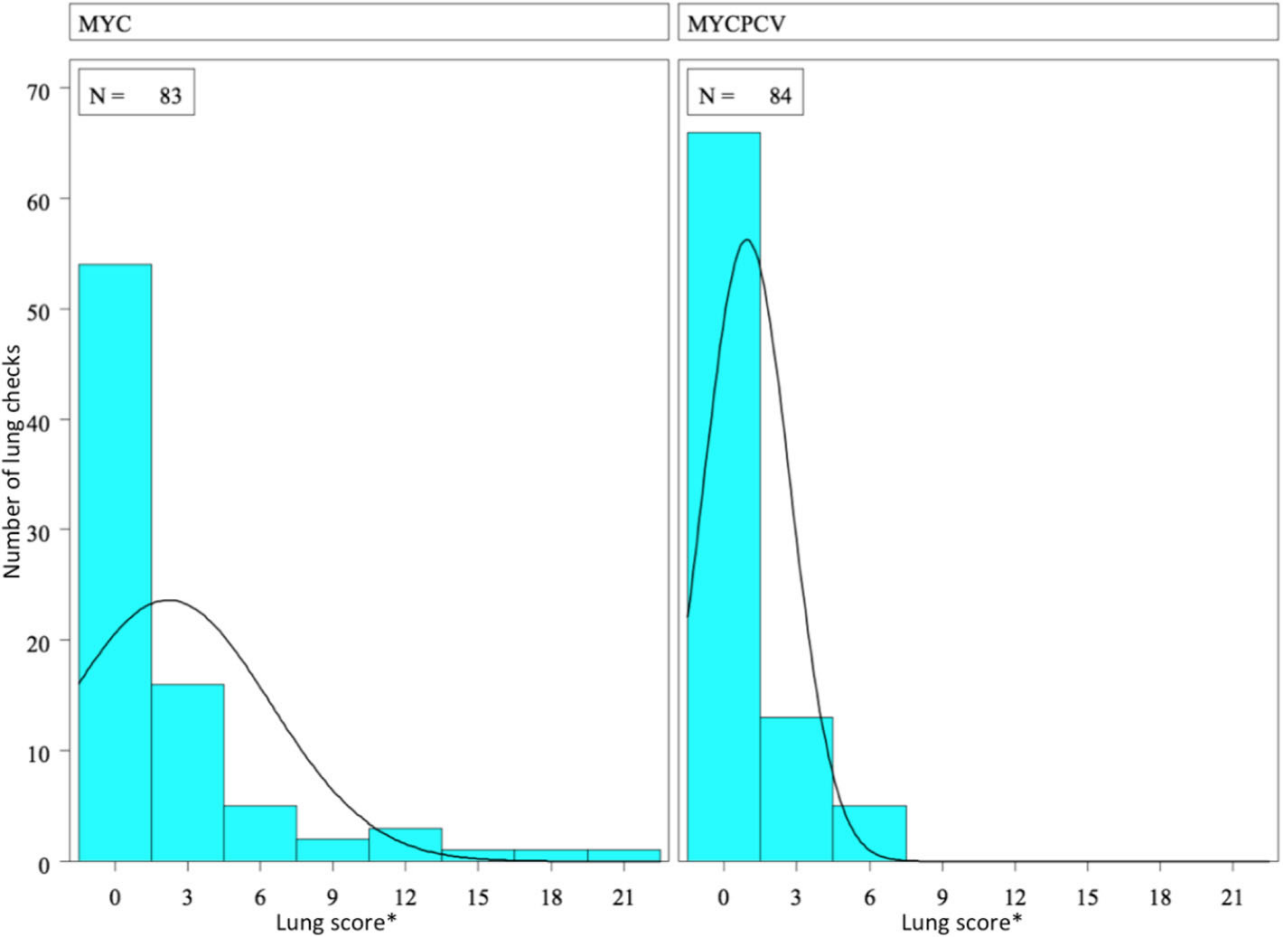
Distribution of the lung lesion scores at slaughter in both trial groups

Lung scoring was performed at the slaughterhouse for the 84 pairs of pigs. The average LLS was lower in the MycPCV group than in the Myc group (0.9 vs. 2.2, respectively), but this difference was not statistically significant (*p* = 0.09, non-parametric Wilcoxon test). However, the proportion of pigs with a LLS ≤ 4 was significantly higher in the MycPCV group than in the Myc group (94 vs. 84%, respectively, *p* = 0.04, Chi-2 test). In addition, no pig in the MycPCV group had a score over 7, whereas 10% of pigs in the Myc group did (see Fig. 6).

## Discussion

The significant difference in ADG over fattening in the group vaccinated against both PCV2 and *M. hyopneumoniae*, as compared to the group vaccinated against *M. hyopneumoniae* alone is consistent with results of a recently published Spanish study on the efficacy of piglet PCV2 vaccination in farms with and without overt clinical signs of PCV2-associated diseases [4]. In both farms, ADG was significantly improved by vaccination during the fattening stage, but not over the post-weaning period. In a Canadian trial where only PCV2 vaccination was performed at weaning (at 3 weeks of age, against placebo), ADG was found to be greater (*p* < 0.01) in the vaccinates than in the controls over the entire study period, even though a clinical ileitis break occurred between finishing days 57–70 in both groups [2]. Also, the level of ADG improvement over finishing (940 in vaccinates vs. 904 g/d in controls, p < 0.01) was found to be comparable to that in the present study (36 and 38 g/d). However, the Canadian herd had a high-health status, with higher growth performance levels than in our trial. In our study design, piglets of the two groups were not commingled in pens, but allocated to separate pens. This might hamper the distinction between the pen-effect and the group-effect. However, the farm in the study was built recently and all pens had an identical structure and equipment; the only difference being the symmetrical disposition around the alley. As the groups were alternately distributed on either side of the alley, it is highly likely that the pen-effect on the measured results is at most limited, if not negligible.

Risk of *M. hyopneumoniae* seropositivity increases with age and production system [11]. In our study, pigs from both groups had comparable serological profiles, with a continuous antibody level decline after week 12. This is suggestive of a vaccine-related seroconversion, rather than one triggered by field challenge.

PCV2 seroconversion took place between 12 and 16 weeks of age, as a result of exposure to the virus, with a marked increase later on. In the MycPCV group, vaccination was followed by a steady increase in PCV2 antibodies until week 12, followed by a drop in week 16. The subsequent seroconversion might be interpreted as the consequence of a delayed PCV2 circulation, due to ‘herd immunity’. Differences in levels of PCV2 viremia between both groups confirm that non-PCV2-vaccinated pigs develop a significantly higher viremia and for a longer duration than vaccinated ones. The viremia levels observed in the Myc group (5.6 log10 genome copies/ml serum) were consistent with the ‘high’ viral load in pigs from a Spanish study of two 5000-sow farms that had a strongly decreased ADG between 3 to 21 weeks [12].

The shorter and lower viremia, the improved ADG, the tendency to an improved homogeneity and an absence of PCV2-like pathological lesions in the lymph nodes of the necropsied PCV2-vaccinated pigs support the protective efficacy of PCV2 vaccination in the face of a subclinical PCV2 infection in a conventional pig farm in Western France.

In terms of economic benefit, age at slaughter was significantly improved by 4.7 days (*p* < 0.05, Table 1). Also, a cost simulation was performed with the online tool designed by IFIP,^3^ which calculated that, for a given 196-sow French farrow-to-finish farm under a 7-week management, the improvement of the fattening ADG by 34 g/d corresponded to €1.7 extra revenue per slaughtered pig (€39 additional revenue per sow per year). This calculation takes into account the higher price of the combination vaccine (as compared to the one-shot *M. hyopneumoniae* vaccine). This estimate seems conservative, even for the year of the trial. A British model estimated that each sub-clinically infected (unvaccinated) pig that reaches slaughter represents a mean loss of €9.52 (90% confidence interval: €2.56–17.75, Alarcon, 2013^4^), at a time when price for live swine was comparable between France and UK.

## Conclusion

The trial was conducted in a farm with housing conditions and health status reflecting those of conventional facilities in Western France. The growth and reproductive performance levels of the farm are in the top-third of the French farrow-to-finish farms. Piglets were routinely vaccinated at weaning (4 weeks of age) with a one-shot *M. hyopneumoniae* bacterin vaccine. The study shows that, compared to that vaccine, the use of Porcilis® PCV M Hyo had no detrimental effect on growth performance post-weaning. It also significantly increased the growth performance during fattening (+34 g/d), while decreasing the age at slaughter by 5 days. These results led to an increase of the net profit by at least €1.7 per slaughtered pig (€39 per sow per year), including the extra-cost of the bivalent vaccine.

Improved FCR and homogeneity of pig batches were also observed, although the changes were not significant, possibly because of the limited number of pigs in the trial (*n* = 140), due to the automatic feeding system constraints. The positive outcome observed in the MycPCV group suggests an improved control of PCV2 infection (lower viremia, of shorter duration, absence of PCV2-like lesions) through vaccination with Porcilis® PCV M. Hyo.

## Abbreviations

ADG: Average daily gain
AUC: Area under the curve
EU: European Union
FCR: Feed Conversion Rate
GBP: British Pound
LLS: Lung lesion score
PCR: Polymerase Chain Reaction
PCV2: Porcine Circovirus type 2
PRRS: Porcine Reproductive and Respiratory Syndrome
PRRSV: Porcine Reproductive and Respiratory Syndrome Virus
SIV: Swine Influenza Virus

## Footnotes

1 These references are available online for 2015 and previous years, in French, at: http://www.ifip.asso.fr/fr/resultats-economiques-gttt-graphique.html

2 Sixteen pigs died during the trial: 10 in the MycPCV group and 6 in the Myc group (no significant difference, exact Fischer test). This higher than usual mortality was associated with heat stress (gastric ulcers were the cause of 63% of deaths): a heat wave occurred during fattening.

3 This tool is freely available at: http://pigsim.com/EN/Presentation/Pages/default.aspx

4 This estimate was expressed in GBP in the original article, with 8.1 GBP mean loss per subclinically infected pig to reach slaughter.
